# The COVID-19-Related Lockdown in Qatar: Associations Among Demographics, Social Distancing, Mood Changes, and Quality of Life

**DOI:** 10.1007/s11469-021-00536-9

**Published:** 2021-05-11

**Authors:** Ahmed M. Megreya, Robert D. Latzman, Aisha M. Al-Ahmadi, Nasser F. Al-Dosari

**Affiliations:** 1 College of Education, Qatar University, P.O. 2713, Doha, Qatar; 2 Department of Psychology, Georgia State University, Atlanta, GA USA

**Keywords:** COVID-19, Demographic variables, Acceptance of social distancing, Mood changes, Quality of life

## Abstract

The worldwide spread of the coronavirus disease (COVID-19), the absence of medical treatment and vaccination, the delayed onset of symptoms, and the rapid human-to-human transmission have led the vast majority of countries to impose strict social distancing procedures. Whereas it appears that social distancing is an effective strategy for mitigating spread, it may also result in a variety of unintended negative consequences to individuals’ psychological well-being and mental health. During the peak of the COVID-19 pandemic, the present study examined associations among some demographic variables (gender, age, marital and working statuses, and having a family member or a friend infected with COVID-19), acceptance of social distancing, mood changes, and quality of life (QoL) in Qatar, a high-income Middle Eastern Arabic-speaking country. Older, married, and working participants were more accepting of social distancing than younger, unmarried, and non-working participants, respectively. Participants indicated that, during this time, they became more distressed, upset, scared, irritable, nervous, and afraid, and less inspired and determined. In a stark contrast, more individuals indicated that they became more interested, alert, and attentive, whereas higher percentages of participants reported feeling less guilty, hostile, and ashamed. Social distancing correlated positively with negative affect, whereas social avoidances correlated positively with positive affect and with physical, psychological, social, and environmental QoL. Finally, positive affect correlated positively, and negative affect correlated negatively, with these four domains of QoL. These results highlight the need for public health and clinical providers to consider peoples’ psychological well-being and mental health during the COVID-19 pandemic.

The worldwide spread of the coronavirus disease (COVID-19), the absence of medical treatment and vaccination, the delayed onset of symptoms, and the rapid human-to-human transmission have led the vast majority of countries to impose a range of different community containment procedures. These interventions range from social distancing (e.g., virtual schooling, working from home, the closure of public markets, the cancelation of social events and trips, voluntary curfew, and the suspension of gatherings) to cordon sanitaire (e.g., mandatory home curfew and locking down an entire city or province); for a discussion of these procedures, see Wilder-Smith and Freedman (2020). Preliminary evidence suggests that the limitation of the unnecessary face-to-face social contacts has been an effective intervention for reducing the spread of the COVID-19 epidemic (Anderson et al., 2020; Betsch, 2020). For example, Betsch et al. (2020) argued that the fast and massive behavioral changes (e.g., social distancing procedures) are the keys for mitigating the COVID-19 crisis, and also note the importance of investigating the psychological and societal consequences as function of individuals’ feelings and perceptions of the crisis.

Whereas during the early stages of COVID-19 it appears that individuals generally accepted and respected social distancing procedures (e.g., see Oosterhoff & palmer, 2020; Rieger, 2020), social distancing might have challenges on individuals’ psychological well-being and mental health, regardless of whether it is voluntary or mandatory (Cao et al., 2020; Marroquín et al., 2020; Tull et al., 2020; Zhang et al., 2020). Notably, whereas the literature on psychological effects of social distancing has ballooned, the overwhelming majority of this work has focused on the Western and Eastern countries. Thus, the present study examined associations among some demographic variables (gender, age, marital and working statuses, and having a family member or a friend infected with COVID-19), acceptance of social distancing, mood changes, and quality of life (QoL) in Qatar, a high-income Middle Eastern Arabic-speaking country.

## Mental Health During COVID-19 Pandemic

An increasingly large number of studies have consistently reported a range of mental health problems during the application of voluntary social distancing procedures. For example, in a review of 19 studies with a total of 93,569 participants from eight different countries (China, Spain, Italy, Iran, USA, Turkey, Nepal, and Denmark), Xiong et al. (2020) demonstrated that mental health–related problems during COVID-19 were more common among females, individuals under 40 years, and people with chronic diseases and a history of medical/psychiatric illnesses. Xiong et al. (2020) further noted that it appeared that frequent exposure to social media/news relating to COVID-19 was a particularly meaningful contributor to symptoms of anxiety and stress, and that lower economic status, lower education level, and unemployment were risk factors for developing symptoms more generally, and depressive symptoms more specifically.

Individual studies across various countries support these conclusions. In Western countries, such as the USA, participants showed a high level of depressive symptoms, exceeding established clinical cutoffs, with more than 25% of one sample reporting moderate to severe anxiety symptoms (Fitzpatrick et al., 2020). In the UK, research has reported increased rates of a range of mental health problem including depression, stress and anxiety, paranoia, hallucinations, and compulsive buying (Lopes & Jaspal, 2020). In Australia, 78% of one sample reported meaningful worse mental health; more than half of participants reported elevated levels of depression (62%), anxiety (50%), and stress (64%); and 25% reported elevated health anxiety in the past week (Newby et al., 2020). In Italy, 57.1% of adults experienced poor sleep quality, 32.1% reported clinically significant levels of generalized anxiety symptoms, 41.8% experienced psychological distress, and 7.6% reported relevant PTSD symptomatology linked to the COVID-19 (Casagrande et al., 2020).

Research suggests that people in Eastern countries are experiencing similar mental health concerns as a result of the pandemic. For example, in China, more than 50% of participants reported moderate-to-severe psychological symptoms including avoidance, intrusion, and hyper-arousal, with females and students experiencing worse outcomes including higher levels of stress, anxiety, and depression (Wang, H. et al., 2020). In India, 33.2% participants reported psychological distress (Varshney et al., 2020). In Bangladesh, approximately 15 to 18% of a sample of university students reported experiencing severe depression and anxiety, respectively, during the lockdown (Islam et al., 2020).

Many fewer studies have been done in Arab-speaking Middle Eastern countries. For example, participants from the Kingdom of Saudi Arabia reported high prevalence rates of anxiety, depression, and stress (40.5%, 57.5%, and 55.5%, respectively); as compared to males, females experienced higher levels of anxiety but lowers level of depression (El Keshky et al., 2020). In Egypt, El-Zoghby et al. (2020) found that roughly 40% of participants showed severe symptoms of intrusion, avoidance, and hyper-arousal during COVID-19 pandemic. In Jordon, Naser et al. (2020) reported prevalence rates of 23.8% and 13.1% for depression and anxiety (respectively) during the peak of COVID-19. In Kuwait, 53.7% and 59.6% of participants experienced anxiety and depression, respectively (Alsharji, 2020). Therefore, considering cross-cultural differences in the impact of COVID-19 pandemic on mental health (e.g., see Dean et al., 2021; Ruiz et al., 2021) and the paucity of studies conducted in Arabic-speaking countries, additional research is needed to enhance our understanding of the impact of the COVID-19 pandemic globally.

## Demographics and Mental Health During COVID-19 Pandemic

Previous studies have identified a variety of demographic factors as risk factors for mental health difficulties during the COVID-19 pandemic. For example, Verma and Mishra (2020) reported that employed participants were two times more depressed and anxious than unemployed participants and that males were more than two times more likely to be anxious than females. As compared to married participants, unmarried participants reported elevated levels of psychological distress (Wang et al., 2020, b). Furthermore, the European Foundation for the Improvement of Living and Working Conditions (Eurofound) project found that younger adults and those out of work had the lowest levels of well-being and that women were less optimistic about their future than men. Further, across a number of studies, age, gender, socio-economic status, and being a parent have emerged as risk factors for adopting more maladaptive coping responses during COVID-19 pandemic (e.g., Atchison et al., 2020; Park et al., 2020). For example, Volk et al. (2021) found that higher income and higher number of children were associated with higher levels of seeking socioemotional and higher avoidance, respectively. Xiong et al. (2020) additionally reported that female gender, chronic diseases, histories of medical or psychiatric illnesses, lower economic status and education level, and unemployment were risk factors for mental health problems generally, and for depression, specifically.

## Emotions During COVID-19

A growing body of research suggests that COVID-19 may be associated with a range of negative emotions (Garbe et al., 2020; Musche et al., 2020; Pérez-Fuentes et al., 2020; Wang et al., 2020, b). For example, Pérez-Fuentes et al. (2020) found perception of threat from COVID-19 correlated positively with sadness-depression, anxiety, anger-hostility, and negative affect, but negatively with joy and positive affect. Within a sample of Chinese participants during the early stages of COVID-19, Wang et al. (2020, b) asked participants to rate how often they have felt a range of emotions. Participants reported elevated levels of psychological distress, and this particularly pronounced among unmarried participants and those who used negative copying styles. Consistently, Musche et al. (2020) found that both cancer patients and healthy controls experienced elevated COVID-19-related fear, distress, and general anxiety. In addition, Garbe et al. (2020) found that participants with higher emotionality reported more perceived threat of COVID-19.

Importantly, however, COVID-19 might be also associated with positive emotions. For example, Moroń and Biolik-Moroń (2021) examined associations among trait emotional intelligence and positive versus negative affective states during the first full week of the lockdown in Poland. Trait emotional intelligence correlated positively with positive affect but negatively with negative affect. When specific emotions were examined, however, a more complicated pattern of results emerged. Specifically, participants reported increases in a range of positive (happiness and relaxation) and negative (anger, anxiety, and sadness) affective states. Interestingly and unexpectedly, positive emotions of relaxation and happiness were experienced more frequently and more intensely as compared to the negatively balanced emotions (Moroń & Biolik-Moroń, 2021). Similar findings have been reported by Ma and Wang (2021) who asked participants to rate how much they experience a range of positive versus negative emotions in response to COVID-19. Results revealed that participants experienced positive emotions more frequently than negative ones. Further, individuals who excessively share negative emotions of others reported experiencing increased depression, anxiety, and stress together with decreased experiences of vigor and other positive emotions. In addition, El-Zoghby et al. (2020) found more than half of the participants felt apprehensive, horrified, and helpless due to COVID-19 pandemic, which also increased caring for family members’ feelings in two-thirds of sample.

Previous research has shown that positivity appears to mediate the association between COVID-19-related perceived risk, death distress, and happiness (Yıldırım & Guler, 2021). Indeed, more optimistic people appear to consider the risk for COVID-19 lower for themselves than for others (Monzani et al., 2021). Importantly, not only does there appear to be variation in the experience of negative psychological outcomes as a result of the pandemic, but also some studies suggest that the vast majority of people have fared quite well. For example, Somma et al. (2020) found that emotion-related problems affected only 13.2% of their sample of community-dwelling adults during the first month of the social distancing period due to the COVID-19 pandemic in Italy; the vast majority of participants (86.8%) endorsed relatively good emotional well-being.

## QoL During COVID-19

In addition to impacts on mental health and emotional well-being, not surprisingly the COVID-19 pandemic appears to be affecting peoples’ QoL. For example, as noted above, the Eurofound project has investigated the impact of COVID-19 on well-being, work, and the financial situation of people across the European Union in two rounds (during the full lockdown in April and the slowly re-opening in July). Initial results of this project suggest that, during the lockdown, individuals have experienced widespread emotional distress, financial concern, and low levels of trust in institutions. Specifically, younger adults and those out of work reported the lowest levels of well-being. While life satisfaction and optimism increased in the second round, younger people continued to feel excluded from society and remained at the greatest risk for depression. In addition, round one data suggested that women were less optimistic about their future than men and this gap was further widened in the second round. Consistent with this report, Pieh et al. (2020) examined associations among relationship quality and a range of domains of QoL in Austria. These authors reported that individuals with good relationship quality reported better QoL across all domains as compared to individuals with poorer relationship quality or without a meaningful relationship; interestingly, individuals without a relationship reported better QoL across domains than individuals with poor relationship quality. Furthermore, Korkmaz et al. (2020) found that QoL correlated negatively with anxiety in a sample of health care workers who were working in COVID-19 outpatient clinics or emergency departments in Turkey; these results did not differ as a result of gender or marital status.

## Current Study

A growing research literature has demonstrated that the COVID-19 pandemic is associated with a range of clinically relevant mental health concerns (for a review, see Xiong et al. (2020). Among many potential demographic contributors, previous studies have found that gender, age, marital and working statuses, and personal experiences with infected cases are significant risks to mental health (e.g., see Atchison et al., 2020; Park et al., 2020. Verma & Mishra, 2020; Volk et al., 2021; Xiong et al., 2020; Wang et al., 2020, b). In addition, the pandemic has been found to associate with increased levels of some negative emotions such as sadness-depression, fear, distress, anxiety, and anger-hostility and surprisingly, also, with increased levels of positive affect including happiness and relaxation (Garbe et al., 2020; Moroń & Biolik-Moroń, 2021; Musche et al., 2020; Pérez-Fuentes et al., 2020; Wang et al., 2020, b). Research to date, however, is not unequivocal with some studies finding that the COVID-19 pandemic might not influence emotional well-being (Somma et al., 2020). Furthermore, although COVID-19 might negatively impact QoL (e.g., Ahrendt et al., 2020), to date, the relationship between mood changes and QoL during the pandemic is still unknown.

Therefore, the aim of the current study was thus three-fold. The first objective was to examine the associations between demographic variables (i.e., gender, age, marital and working statuses, and having a family member or a friend infected with COVID-19) and acceptance of social distancing procedure and QoL during the peak of the pandemic and related lockdown procedure in the high-income Middle Eastern Arabic-speaking country of Qatar. The second aim was to investigate the impact of lockdown procedures related to COVID-19 on changes in mood. Finally, associations among social distancing, mood changes, and QoL were investigated.

## Method

### Participants

A convenience sample of 280 undergraduate and post-graduate students from Qatar University volunteered to participate in this study. Participants’ age ranged from 17 to 53 years, with a mean of 27.3 years (SD = 8.6). 60.7% were females and 39.3% were males. Ninety percent were undergraduate students and 10% were post-graduates. 39.6% were working and 60.4% were not working. 54.3% were married while 41.4%, .03%, and .01% were unmarried, divorced, or widowed, respectively. 70.4% were Qataris and 29.6% were non-Qataris. 22.5% of the sample had a family member with COVID-19 and 70.4% had a friend with this condition. .05%, 61.1%, 29.3%, and .05% of the participants reported low, medium, high, and very high monthly income, respectively. Ethical approval for participation in this study was provided by Qatar University’s institutional review board (QU-IRB) and all procedures were in accordance with QU-IRB guidelines and regulations. Informed consent was obtained from all participants before they had given the questionnaires.

### Measures

#### Acceptance of Social Distancing

The *COVID-19 Snapshot Monitoring* (COSMO; Betsch et al., 2020) survey, which has been adapted by the WHO Regional Office for Europe (2020), includes two subscales that quantify the acceptance of a range of social distancing procedures including avoiding social contacts, crowded areas, and foreigners and canceling travels. The first subscale is a part of a variable entitled “preparedness – barriers and drivers” that includes five social distancing items, using a 7-point ranking scale ranging from 1 (strongly disagree) to 7 (strongly agree). The other subscale is a part of a variable entitled “panic buying / panic behavior” that includes eight items to indicate foreigners and social event avoidances and travel cancelations using a 3-point rating scale (1 = done, 2 = planned, and 3 = not done nor planned). Items, along with descriptive statistics, are presented in Table 2. In the present study, alpha reliability rates were 0.74 and 0.77 for these two subscales, respectively. The COSMO has been translated into several languages, including Arabic, and has been used in several countries (WHO Regional Office for Europe, 2020).

#### Quality of Life

The *World Health Organization Quality of Life Brief Questionnaire* (WHOQOL-BREF; WHO, 1996) is a brief 26-item version of the WHOQOL-100 (WHO, 1996) that consists of 24 items assessing four main domains of QoL. These domains include physical health (8 items), psychological health (6 items), social relationships (3 items), and environment (7 items). Additionally, there are two items describing the overall QoL and general health of the respondent. Using a 5-point Likert rating scale, participants are required to describe how they feel about your QoL, health, or other areas of your life in the last two weeks. Higher scores indicate higher levels of QoL. To meet the requirements of our local IRB committee, item numbered 21 (“How satisfied are you with your sex life?”) was removed prior to administration. Accordingly, the domain of Social Relationships involved only two items in the present study. The domain scores of the WHOQOL-BREF were calculated using means of rating scores. The WHOQOL-BREF has been translated into many different languages, including Arabic, and the psychometric proprieties across all of these translations were good to adequate (Skevington et al., 2004). The factorial structure of the Arabic version has been confirmed in different Arab countries such as Sudan (Ohaeri et al., 2007), Saudi Arabia (Ohaeri & Awadalla, 2009), and Kuwait (Ohaeri et al., 2009), with good reliabilities rates. In the present study, alpha reliability rates were .80, .86, .79, and .88 for the four domains, respectively.

#### Mood

The *Positive and Negative Affect Schedule* (PANAS: Watson et al., 1988) is a 20-item measure of positive and negative affect. Each item consists of one adjective word describing a specific positive (e.g., excited, enthusiastic, and alert) or negative (e.g., afraid, distressed, and upset) emotions. The original scale requires participants to indicate to what extent they have generally experienced the 20 emotional states using a 5-point Likert-type scale ranging from 1 (*very slightly or not at all*) to 5 (*extremely*). The Arabic version of the PANAS has been validated and replicated the adequate psychometric proprieties of the original scale (Davis et al., in press; Megreya et al., 2016; Megreya et al., 2018). In the present study, the PANAS was modified to enable participants to decide whether each emotion is increased (scored +1), decreased (scored −1), or unchanged (scored 0) during the COVID-19. Therefore, the −1 to +1 scores were used to create scales of changes in positive and negative affective states, which were then used for subsequent analyses. The alpha reliability rates of this modified version were .76 and .80 for positive affect and negative affect, respectively.

### Procedure

Following a public announcement to all Qatar University students, data were collected online using Google Forms during a one-month period from 11 June 2020 to 11 July 2020. All who participated were presented with the IRB consent form, which was ended by a declaration of accepting participation in this study. After completing the demographic information questionnaire, participants completed the *COSMO Acceptance of Social Distancing questionnaire*, *PANAS*, and WHOQOL-BREF questionnaires, in this order.

### Statistical Analyses

Descriptive statistics (means, 95% confidence intervals, and standard deviations) were reported for each variable, and alpha Cronbach method was used to examine the reliability of each instrument. In addition, Pearson correlation coefficients were used to examine the correlations between some demographic variables (age, marital status, working status, and having a family member or a friend infected with COVID-19) and the acceptance of social distancing and QoL, and between affect changes and QoL. Furthermore, chi-square goodness-of-fit was utilized to examine the differences among participants who had reported that their positive and negative emotions were increased, decreased, or not changed related to the COVID-19.

## Results

### Acceptance of Social Distancing

Table 1 presents descriptive statistics for the COSMO subscales of the Social Distancing Survey. Social distancing was correlated positively with age, *r* (278) = .26, *p* < 0.001, but negatively with marital status (with married, non-married, divorced, and widow were coded as 1, 2, 3, and 4, respectively), *r* (178) = −.29, *p* < 0.001, and work (with working and none-working were coded as 1 and 2, respectively), *r* (178) = −.26, *p* < 0.001. These results indicate that older, married, and working participants accepted social distancing more highly than others did. However, accepting social distancing did not correlate with gender, *r* (178) = .07, *p* = 0.258, income, *r* (178) = −.03, *p* = 0.588, whether or not having a family member, *r* (178) = .07, *p* = 0.274, or a friend, *r* (178) = .02, *p* = 0.708, infected with COVID-19. The other subscale “social avoidances” did not correlate with any demographic variables nor with the first subscale, *r* (178) < 1 or − 1.

**Table 1:** Descriptive statistics for the acceptance of social distancing subscales

COSMO subscales	*M* (95% CI)	SD (95% CI)	Minimum	Maximum
Subscale 1 (social distancing)	6.04 (5.93 to 6.16)	1.02 (.87 to 1.16	1	7
My family and friends avoid crowded areas	5.97 (5.81 to 6.12)	1.39 (1.24 to 1.55)	1	7
My family and friends avoid social contacts	5.40 (5.20 to 5.59)	1.67 (1.52 to 1.81)	1	7
Health authorities urge me to avoid crowded areas	6.72 (6.61 to 6.82)	.86 (.61 to 1.07)	1	7
I want to protect others by avoiding crowded areas	6.47 (6.34 to 6.60)	1.13 (.94 to 1.32)	1	7
My employer urges me to avoid crowded areas at work (if applicable)	5.66 (5.43 to 5.89)	1.96 (1.77 to 2.13)	1	7
Subscale 2 (social avoidances)	2.47 (2.41 to 2.53)	.49 (.44 to .54	1	3
Avoided people who come from countries where corona virus cases have occurred	2.54 (2.46 to 2.62)	.69 (.63 to .74)	1	3
Stayed away from social events I had planned to attend	2.70 (2.62 to 2.77)	.61 (.53 to .67)	1	3
Canceled flights or train rides	2.53 (2.43 to 2.63)	.80 (.72 to .85)	1	3
Canceled holiday trips	2.63 (2.54 to 2.71)	.74 (.66 to .80)	1	3
Canceled business trips	2.38 (2.28 to 2.49)	.89 (.83 to .93)	1	3
Avoided visiting family even when I did not have symptoms of disease	2.45 (2.36 to 2.55)	.78 (.72 to .83)	1	3
Asked family members or friends not to visit me	2.20 (2.10 to 2.43)	.87 (.83 to .90)	1	3
Decided that my child could not meet with a friend	2.32 (2.22 to 2.43)	0.91 (.86 to .94)	1	3

### Changes in Affective States

**Fig. 1:**
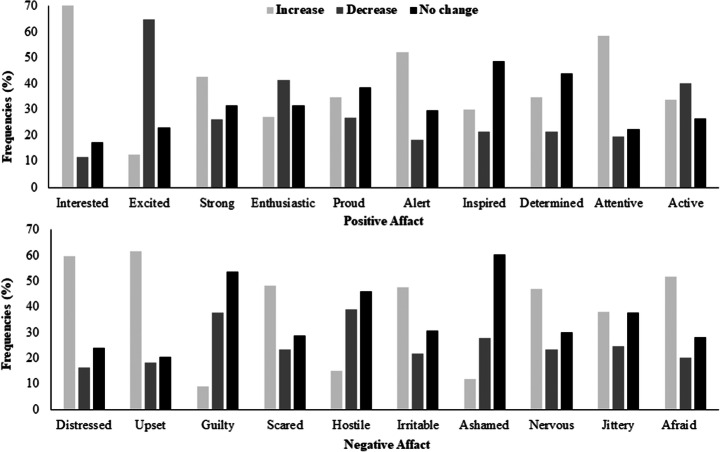
Percentages of the frequencies of participants who had reported that their positive versus negative emotions were increased, decreased, or not changed

Table 2 shows descriptive statistics of the changes in affective states related to COVID-19, with positive scores indicating increments while negative scores indicating decrements. Figure 1 shows the percentage frequencies of participants who had reported that their positive and negative emotions were increased, decreased, or not changed related to the COVID-19. Table 3 shows the results of chi-square goodness-of-fit that indicated significant differences among the three groups of participants (who decided that their emotions were increased, decreased, or not changed) in all positive and negative emotions. Importantly, more individuals indicated that they became more *interested*, *alert*, *attentive*, *distressed*, *upset*, *scared*, *irritable*, *nervous*, and *afraid*, whereas higher numbers of participants pointed out to be less *inspired*, *determined*, *guilty*, *hostile*, and *ashamed* (see Fig. 1).

**Table 2:** Descriptive statistics of the modified version of PANAS

	*M* (95% CI)	SD (95% CI)	Minimum	Maximum
Positive affect	.11 (.06 to .15)	.43 (.39 to .46)	-1	1
Interested	.60 (.51 to .68)	.69 (.61 to .75)	-1	1
Excited	−.52 (−.60 to −.43)	.71 (.65 to .76)	−1	1
Strong	.16 (.06 to .26)	.81 (.77 to .85)	−1	1
Enthusiastic	−.14 (−.24 to −.04)	.82 (.78 to .85)	−1	1
Proud	.08 (−.01 to .17)	.78 (.74 to .81)	−1	1
Alert	.34 (.25 to .43)	.77 (.72 to .81)	−1	1
Inspired	.09 (.01 to .17)	.71 (.67 to .75)	−1	1
Determined	.13 (.05 to .21)	.74 (.69 to .78)	−1	1
Attentive	.39 (.30 to .49)	.79 (.74 to .84)	−1	1
Active	−.06 (−.16 to .03)	.86 (.82 to .88)	−1	1
Negative affect	.14 (.08 to .19)	.44 (.41 to .47)	−1	1
Distressed	.43 (.34 to .52)	.76 (.70 to .80)	−1	1
Upset	.43 (.34 to .53)	.78 (.73 to .83)	−1	1
Guilty	−.29 (−.36 to −.21)	.62 (.57 to .66)	−1	1
Scared	.25 (.16 to .35)	.81 (.76 to .84)	−1	1
Hostile	−.24 (−.32 to −.16)	.69 (.65 to .74)	−1	1
Irritable	.26 (.17 to .35)	.79 (.75 to .83)	−1	1
Ashamed	−.16 (−.23 to −.08)	.61 (.56 to .65)	−1	1
Nervous	.24 (.15 to .32)	.80 (.76 to .84)	−1	1
Jittery	.13 (.04 to .22)	.78 (.74 to .82)	−1	1
Afraid	.32 (.22 to .41)	.79 (.74 to .83)	−1	1

**Table 3:** Results of chi-square goodness-of-fit for the frequencies of mood changes

Affect	*χ*^2^ df = 2	*p*
Interested	184.23	0.000
Excited	128.02	0.000
Strong	11.79	0.003
Enthusiastic	9.03	0.011
Proud	6.05	0.049
Alert	50.06	0.000
Inspired	32.34	0.000
Determined	21.48	0.000
Attentive	80.6	0.000
Active	7.74	0.02
Distressed	89.58	0.000
Upset	99.65	0.000
Guilty	85.89	0.000
Scared	29.11	0.000
Hostile	44.49	0.000
Irritable	28.64	0.000
Ashamed	102.86	0.000
Nervous	24.74	0.000
Jittery	9.52	0.000
Afraid	45.74	0.000

### Quality of Life

Table 4 presents descriptive statistics for the QoL domains. Pearson correlation coefficients showed no correlation between the six indictors of QoL and demographics (age, gender, working and marital statuses, income, and having a friend or a family member infected with COVID-19), all *r*s < 1 or − 1, except a negative correlation between Social Relation domain and age, *r* (178) = −.12, *p* = .048. Table 5 shows inter-correlations among the WHOQOL-BREF subscales. There were strong positive inter-correlations among all subscales, mean *r* = .57, *p* < .001.

**Table 4:** Descriptive statistics of QoL

	*M* (95% CI)	SD (95% CI)	Minimum	Maximum
Overall QoL	3.90 (3.78 to 4.02)	0.98 (.89 to 1.05)	1	5
General health	4.05 (3.93 to 4.18)	0.96 (.86 to 1.05)	1	5
Physical health	3.38 (3.30 to 3.47)	0.72 (.66 to .78)	1.13	5
Psychological	3.54 (3.45 to 3.64)	0.81 (.74 to .87)	1.33	5
Social relations	3.73 (3.61 to 3.84)	0.98 (.89 to 1.06)	1	5
Environment	3.70 (3.60 to 3.80)	0.86 (.80 to .92)	1	5

**Table 5:** Inter-correlations among the WHOQOL-Bref subscales

	Overall QoL	Overall health	Physical	Psychological	Social
Overall health	.493**				
Physical	.543**	.516**			
Psychological	.662**	.560**	.754**		
Social	.369**	.362**	.572**	.607**	
Environment	.647**	.494**	.657**	.727**	.586**

*QoL* quality of life; **p < 0.001

### Associations Among Social Distancing, Mood Changes, and QoL

Table 6 shows Pearson correlation coefficients among acceptance for social distancing, mood changes, and QoL. To summarize, social distancing correlated positively with negative affect (*r* = .12), but it did not correlate with positive affect and all QoL indictors. On the other hand, social avoidances correlated positively with positive affect (*r* = .25) and all QoL indicators (mean *r* = .13) except overall health but it did not correlate with negative affect. Positive affect correlated positively with all QoL indicators (mean *r* = .41) whereas negative affect correlated negatively with all of these indictors (mean *r* = −.34).

**Table 6:** Correlations among social distancing, positive/ negative affect and QoL

	Social distancing	Social avoidances	Positive affect	Negative affect
Social avoidances	−.04			
Positive affect	−.08	.25**		
Negative affect	.12*	−.04	−.43**	
Overall QoL	−.01	.13*	.34**	−.33**
Overall health	−.03	.11	.44**	−.29**
Physical health	.06	.13*	.51**	−.43**
Psychological	−.04	.14*	.48**	−.38**
Social relationships	−.03	.12*	.35**	−.29**
Environment	−.07	.15**	.36**	−.33**

## Discussion

During the peak of COVID-19 in a high-income Middle Eastern Arabic-speaking country (Qatar), the present study examined the associations among variations and associations among demographic characteristics, acceptance of social distancing, mood changes, and QoL. A number of interesting findings were revealed that would be discussed in the following sub-sections.

### Demographic Variables

The results of the present study demonstrated that older, married, and currently working participants accepted social distancing more than younger, unmarried, and non-working participants, respectively. However, gender, income, and having a family member or a friend infected with COVID-19 did not correlate with acceptance of social distancing. In addition, no relationship was found between social avoidances and any of the demographic variables. Furthermore, with the exception of a negative correlation between Social Relation QoL domain and age, no relationship was found between QoL and demographic characteristics. These findings are partially consistent with results of previous studies, which reported significant associations between demographic characteristics and perceptions of compliance with social distancing procedures and mental health (e.g., see Atchison et al., 2020; Volk et al., 2021; Xiong et al., 2020). For example, consistent with previous studies, results of the present study suggest that older participants are more accepting of social distancing. This finding could be explained by public health messaging cautioning that the severity COVID-19 symptoms increases with age (WHO, 2021). Consistently, Atchison et al. (2020) found that adoption of social distancing measures was higher in those aged over 70 years compared to younger adults aged 18 to 34 years. However, in contrast to the results of the current study finding that marital status was not associated with QoL and affect changes, previous studies have reported elevated levels of psychological distress among unmarried participants in China (Wang et al., 2020, b). One potential explanation for these differences may be the generally higher level of QoL in Qatar as a high-income country.

Indeed, previous studies have further reported a significant association between income and perception of COVID-19 and mental health (Atchison et al., 2020; Bodas & Peleg, 2020; Volk et al., 2020). For example, Bodas and Peleg (2020) reported that the percentages of compliance with public health regulations dropped from 94% when people in self-quarantine continued to receive their salaries to 57% when there was no compensation for lost wages. In addition, Atchison et al. (2020) found that people with the lowest household income were six times less likely to be able to work from home and three times less likely to be able to self-isolate. Furthermore, Volk et al. (2020) reported that higher income was associated with higher levels of seeking socioemotional support suggesting that financial resources might afford individuals the time and means to connect with those close to them. Along these same lines, Xiong et al. (2020) concluded that, during the pandemic, poor economic status and unemployment are significant risk factors for developing symptoms of mental disorders, especially depressive symptoms. Importantly, however, the present study did not find income to correlate with either acceptance for social distancing nor QoL. One likely explanation for this lack of association in the current study is that, in addition to the generally high-income levels among citizens, people in Qatar did not lose any wages or compensations during social distancing procedures.

### Mood Changes

The present study showed that individuals’ affective states were significantly changed as a result of the COVID-19 pandemic (see Fig. 1). Specifically, participants indicated that they became more *distressed*, *upset*, *scared*, *irritable*, *nervous*, and *afraid*. In addition, participants reported being less *inspired* and *determined*. Consistent with these findings, across a range of different countries, previous studies have reported elevated levels of stress, anxiety, and depression during the lockdown related to COVID-19 (Casagrande et al., 2000; El Keshky et al., 2020; Fitzpatrick et al., 2020; Islam et al., 2020; Lopes & Jaspal, 2020; Newby et al., 2020). Increased levels of negative emotions might be explained by several factors potentially resulting from the pandemic (such as the high prevalence rates of COVID infections and lack of access to treatments), individual-difference level factors (such as perceived stress, personality traits, coping strategies, sudden changes in lifestyles, and work-related worries), and media exposure (such as the dominance of pandemic-related news); for a review, see Xiong et al. (2020). Importantly, however, the subjective unpleasantness of negative emotions, especially anxiety-related symptoms, might also have a positive, more adaptive side related to keeping one safe (e.g., for a review see, Perkins & Corr, 2014). For example, Harper et al. (2020) found that fear of COVID-19 was the most reliable predictor of the public health-compliant behaviors (e.g., social distancing and improved hand hygiene). Accordingly, these authors suggested that negative emotions, especially fear and anxiety, in response to COVID-19 might have an adaptive, functional role (Harper et al., 2020).

The present study found increments in some positive affective states during the lockdown as well. Specifically, individuals indicated that they became more *interested*, *alert*, and *attentive*, whereas higher percentages of participants reported being less *guilty*, *hostile*, and *ashamed* (see Fig. 1). These increases in positive emotions might be explained by several factors. Intuitively, one such factor might be related to the positive influences of staying at home on family relationships. For example, Al-Sabbah et al. (2021) found students from two Arabic countries (Jordon and United Arab Emirates) endorsed a relatively higher ranking (3.61 out of 5) to a self-report item stating that “*My family has a positive effect on me*” In addition, more than 70% of participants agreed that they had more time to communicate with their family during the pandemic in Jordan than they had previously. Importantly, based on Walsh’s (2015) family resilience framework, Prime et al. (2020) suggested that communication, organization, and belief systems with one’s family could serve as significant sources of resilience to the COVID-19 pandemic.

Consistent with the results of this study, Moroń and Biolik-Moroń (2021) reported increments in anger, anxiety, and sadness, but they also noted that happiness and relaxation were increased. More importantly, Moroń and Biolik-Moroń (2021) found that relaxation and happiness were experienced more frequently and more intensely compared to those negative affective states; that is, increases in positive emotions were particularly pronounced when compared to negative emotions. Ma and Wang (2021) also found that participants experienced positive emotions more intensely than negative ones. Therefore, the present findings, along with those reported previously (Ma & Wang, 2021; Moroń & Biolik-Moroń, 2021), suggest that the lockdown-related COVID-19 appear to have changed affective states greatly and in a complicated way with increases in both negative (e.g., fear, anxiety, and distress) and positive (e.g., interest, vigilance, relaxation, and happiness) emotions.

### Social Distancing, Mood Changes, and QoL

The present study found that social distancing correlated positively with negative affect but, interestingly, did not correlate with any of the four domains of QoL. In contrast, social avoidances correlated positively with positive affect and with all four domains of QoL. In addition, positive affect correlated positively with the four domains of QoL, whereas negative affect correlated negatively with all of these domains. Consistent with these findings, Korkmaz et al. (2020) found that anxiety correlated negatively with QoL during the spread of COVID-19. In addition, Harper et al. (2020) reported that the fear of COVID-19 correlated negatively with physical and environmental QoL, but there was no correlation between fear of COVID-19 and psychological and social QoL. Furthermore, during normal circumstances prior to the spread of COVID-19, the four domains of QoL have been found to correlate positively with positive affect and negatively with negative affect (e.g., Medvedev & Landhuis, 2018).

### Limitations and Conclusions

This study has a number of limitations. First, the convenient nature of the study sample (i.e., relatively young, gender imbalanced sample of university students) may limit the generalizability of results. Second, given the cross-sectional nature of the study, data were not available for QoL before and during the spread of COVID-19. Third, a WHOQOL-BREF item assessing satisfaction of sexual life was removed. This was a result of only a minority of participants being married and prohibitions against sex outside of marriage in Qatar. Finally, questionnaires were administered in the same order across all participants giving some chances for an order effect. Nevertheless, the current study has a number of notable strengths. First, data were collected from a relatively large number of participants during the full lockdown that was related to the peak of COVID-19 pandemic in a high-income Middle Eastern Arab-speaking country (Qatar); this represents a particularly highly neglected context within the empirical literature (for a review, see Henrich et al., 2010). Second, we utilized a modified version of the PANAS to assess changes in positive and negative affective states before and during the COVID-19 pandemic. Third, this is the first study to date to examine the impact of social distancing related to COVID-19 on mood changes and to investigate the associations between mood changes and QoL.

All told, limitations, notwithstanding the results of the present study, highlight significant challenges for psychological well-being and QoL during the spread of COVID-19 pandemic and suggest avenues for intervention and potential prevention efforts in the future.
